# High genetic abundance of *Rpi-blb2/Mi-1.2/Cami* gene family in *Solanaceae*

**DOI:** 10.1186/s12862-015-0493-z

**Published:** 2015-09-30

**Authors:** Lina Zhao, Qijun Zhang, Rongchao Gao, Sihai Yang, Haoxuan Liu, Xiaohui Zhang

**Affiliations:** State Key Laboratory of Pharmaceutical Biotechnology, School of Life Sciences, Nanjing University, Nanjing, 210000 China; Jiangsu Academy of Agricultural Sciences, Nanjing, 210000 China

**Keywords:** Plant *R* gene, *Rpi-blb2/Mi-1.2/Cami*, Evolutionary history

## Abstract

**Background:**

Three *NBS-LRR* genes, *Rpi-blb2, Mi-1.2*, and *Cami*, constitute a very special plant resistance gene family. These genes confer resistance against 4 distantly related pathogen species in 3 different *Solanaceae* hosts. To characterize this noted resistance, we conducted a series of studies on this gene family.

**Results:**

First, homologs of this gene family were identified in the pepper, tomato and potato genomes. This revealed a large variation in copy number within this gene family among species and a great divergence was found both between and within species. To gain more information pertaining to gene resistance within this family, 121 LRR regions were cloned in 16 different wild/cultivated potato accessions. Again, frequent copy number variations and a high level of divergence between homolog were observed common among accessions. The divergence within species was so high that it reaches the level of divergence between species. Also, frequent frameshift mutations and abundant gene conversion events were identified in these LRR regions.

**Conclusions:**

Our findings suggest that this family harbors an unusually high level of genetic abundance, making it of particular interest. Together with other reported examples, our study also provides evidence that multi-resistance is a common trait in *R* gene families like this.

**Electronic supplementary material:**

The online version of this article (doi:10.1186/s12862-015-0493-z) contains supplementary material, which is available to authorized users.

## Background

The potato, along with the tomato and pepper, are members of *Solanaceae* family, with the potato being one of the world’s most important food crops [1]. Yet the potato is susceptible to a wide range of pathogens, with potato late blight being the most devastating. Potato late blight is caused by *Phytophthora infestans* [2], which greatly effects potato cultivation. Annual potato production is currently over 300 million tons and potential production could exceed 400 million if potato late blight could be properly controlled [3]. Additionally, late blight disease is also responsible for the European potato famine in the 19^th^ century which almost completely destroyed potato crops and led to the starvation of millions of people [4]. Thus, the control of such plant diseases is of fundamental importance.

Plants have evolved sophisticated systems to recognize pathogenic proteins. These recognition proteins, usually referred to as resistance (*R*) genes [5], work in a highly specific manner according to the gene-for-gene interaction model, in which one plant *R* gene responds to a pathogen carrying a particular avirulence (*Avr*) gene [6]. This places the host *R* gene and pathogen *Avr* gene in a co-evolutionary model in which the *Avr* gene evolves different genotypes to avoid recognition while the *R* genes evolve to recognize the *Avr* genes. Studies have shown that mutations and deletions occur at high frequencies in *Avr* genes, as a response of high rates also noted in *R* genes [6, 7].

During the past years, many plant *R* genes have been identified. Most cloned *R* genes belong to a large gene family and encode proteins with nucleotide-binding sites and leucine-rich repeat (NBS-LRR) domains [8]. In potatoes, *NBS-LRR* genes such as *R1*, *RB*, *R2*, *R3*, *Rpi-bib2*, and *Rpi-vnt1.1* have been associated with late blight resistance [9–15]. Among these genes, *Rpi-blb2* is of particular interest due to its homologs conferring resistance in 3 different species and against at least 4 different pathogens. The first member of the *Rpi-blb2* family found to confer resistance was *Mi-1.2* [14], which provides root knot nematode resistance in tomatoes, with this gene also found to confer aphid resistance [16]. Later, *Mi-1.2* was found to display resistance to sweet potato whitefly [17], making it the first and the only plant *R* gene that confers resistance against three distantly related pathogens. In 2005, the late blight resistance gene *Rpi-blb2* identified in potatoes was found to be a *Mi-1.2* homolog of *Mi-1.2* (81 % amino acid sequence identity). The third *R* gene cloned from this family, *Cami*, is from hot peppers and has been found to confer root knot nematode resistance, with a 99 % amino acid sequence identity shared with *Mi-1.2* [18]. Three genes, *Mi-1.2*, *Rpi-blb2*, and *Cami*, derived from different *Solanaceae* organisms confer resistance against various highly divergent pathogens and have originated from the same gene family, making a common ancestor highly possible. What is it that makes this gene family so special and are there more gene families like this?

To answer these questions, we studied the evolutionary history of this gene family. In brief, all homologs of these three resistance genes are referred to as *Rpi-blb2* homologs hereafter. First, homologs of this gene family across the tomato, potato, and pepper genomes were identified, with a varied copy number (from 22 to 40) and a great nucleotide diversity of this gene family was found within species and between species. Next, the potato LRR regions of this gene family were examined and 121 homologs were cloned from 16 potato accessions. A variation in copy number was common between the potato accessions and a high level of nucleotide divergence was found both between accessions and within accessions, suggesting rapid intraspecies and interspecies evolution in this gene family. Meanwhile, strong positive selection and frequent sequence exchanges were also identified in the LRR regions. Overall, our findings indicate the presence of a fast evolving *R* gene locus with a dramatic variation in copy number, high genetic abundance, and a strong diversifying selection. The above findings provide further insight enabling the identification of more novel *R* genes from this special family.

## Methods

### 1. Genomic source and homolog identification

Three fully sequenced genomes were employed in this study, including genome of pepper (The Pepper Genome Database: http://peppersequence.genomics.cn/page/species/download.jsp), genome of tomato (sol genomics network: http://solgenomics.net/organism/Solanum_lycopersicum/genome) and genome of potato (Potato Genomic Resource at Michigan State University, http://solanaceae.plantbiology.msu.edu/).

The re-annotated *NBS-LRR* gene sets of potato and tomato were downloaded from previous studies [19, 20].

To identify the homologs of Rpi-blb2/Mi-1.2/Cami, the coding sequence (CDS) of these 3 genes were used as query and BLAST against the whole-genome CDSs of the 3 genomes and 2 re-annotated gene sets. The E-value was set at 1e-50 and other parameters used default settings. Hits with > 60 % coverage and > 60 % identity were deemed homologs.

### 2. Cloning of LRR domain of this gene family from potato accessions

Degenerate primers were designed on conserved sites at the edges of the LRR regions in order to gain comprehensive LRR domain sequence information (Additional file 1). PCR was performed in 16 potato accessions/genotypes, including 9 wild accessions (*S. demissum* 343–1, *S. demissum* 585–7, *S. microdonatum* 1169, *S. bulbocastanum* 947–2, *S. bulbocastanum* 947–1, *S. bulbocastanum* 948–5, *S. stoloniferum* 298–1, *PP10* and *S. bulbocastanum* 948–2) and 7 cultivated accessions (*S. tuberosum* cv. K6, *S. tuberosum* cv. 872, *S. tuberosum* cv. 873, *S. tuberosum* cv. Sarpo Mira, *S. tuberosum* cv. G18, *S. tuberosum* cv. dongnong308 and *S. tuberosum* cv. kexin18, see Additional file 2 for source of materials). PCR products were cloned into a PGEM-T Easy Vector and ~20 colonies of each cultivar were then sequenced by ABI3100A automated sequencer until no new homolog sequence could be identified.

Sequences for all cloned LRR sequences have been deposited in the GenBank under accession number from KR106459 to KR106579.

### 3. Sequence alignment and phylogenetic analysis

The CDSs or cloned LRR sequences were first translated into amino acid sequence and aligned by MUSCLE implanted in MEGA5 [21] and then back translated into nucleotide sequences. Based on the alignments, a maximum likelihood tree was constructed. First, jModelTest 2.1.7 [22] was used to test the best fitted nucleotide substitution model for tree construction, then this model was used by PhyML 3.1 [23] with 1000 bootstrap replicates for maximum likelihood tree construction.

The nonsynonymous (Ka) and synonymous (Ks) nucleotide substitutions were calculated by DnaSP version5.0 based on the Nei-Gojobori method [24, 25]. Nucleotide diversity (π) and divergence (Dxy) were estimated by π and Dxy with Jukes and Cantor correction [26] using DnaSP version5.0. Gene conversion events were analyzed by GENECOV1.81 (http://www.math.wustl.edu/~sawyer/geneconv/).

## Results

### 1. Characterization of *Rpi-blb2* homologs in three species

To construct the evolutionary relationship of the three *NBS-LRR* genes *Mi-1.2*, *Rpi-bib2*, and *Cami*, their homologs were first identified in genomes of the 3 species (Table 1). Although the genome size of pepper (3,300 Mb) was significantly larger than tomato (900 Mb) and potato (844 Mb), the annotated gene number of these 3 species was on the same level (34,771 ~ 39,031). It is reported that the genome expansion of the hot pepper is attributed to accumulation of repetitive sequences [27, 28]. More *NBS*-encoding genes have been detected in the pepper reference genome than in the tomato or potato reference genomes (Table 1). Since most *NBS* genes are duplicated genes, it is difficult to assembly and annotate these genes, and studies have showed that a certain number of *NBS* genes were missed in potato and tomato reference genomes [19, 20]. Jupe et al. and Andolfo et al. have identified 755 and 326 *NBS* genes by RenSeq in potato and tomato, respectively [19, 20]. As a result, we identified 29, 22 and 40 homologs in the genomes of potato, tomato and pepper, respectively. After removing incomplete sequences, 23, 18 and 40 homologs from potato, tomato and pepper were used for further analysis.

**Table 1:** Number of *Rpi-bib2* homologs in tomato, pepper and potato genomes

Species	Genome size	Predicted gene no.	NBS gene no.	Number of homologs	π	π (NBS)	π (LRR)	*Ka/Ks*	*Ka/Ks* (NBS)	*Ka/Ks* (LRR)
Tomato	900 Mb	34,771	267 (326*)	22	0.285	0.200	0.339	0.599	0.477	0.711
Pepper	3,300 Mb	34,903	684	40	0.270	0.217	0.328	0.584	0.482	0.688
Potato	844 Mb	39,031	443 (755*)	29	0.246	0.223	0.275	0.644	0.564	0.813

*These data are from re-annotated NBS gene sets by previous studies [19, 20]

The nucleotide diversity (π) and *Ka/Ks* were calculated for the homologs from each genome. The divergence varied a lot between genomes. The homologs from potato genome were found to be the most conservative with the lowest nucleotide diversity (0.246), while the homologs from tomato genome were the most divergent (0.285). Such a huge difference was not found when examining *Ka/Ks* ratios which ranged from 0.584 to 0.644 among the different genomes. The nucleotide diversity and *Ka/Ks* were also determined for the NBS domain and LRR domain separately. Across all of the three genomes, the LRR domain exhibited a higher nucleotide diversity and a higher *Ka/Ks* than the NBS domain (Table 1).

**Fig. 1:**
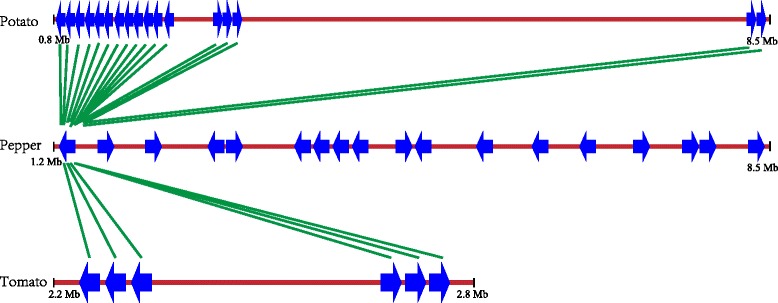
Positional relationships among *Rpi-blb2/Mi-1.2/Cami* homologs from potato, pepper and tomato reference genomes. Genes are represented by blue arrows and genes with highest similarity between species are linked by green lines

Most of the identified homologs were located in tandem on chromosome 6 in each genome. Tandem repeat is a major mechanism in NBS-LRR expansion, with previous studies showing that *NBS-LRR* gene homologues often cluster in tandem on the chromosome [29]. To explore the relation of genomic position among these homologs, a comparative position map of all of the genes on chromosome 6 was constructed (Fig. 1 and Additional file 3), extensive conservation of the gene order between these genomes was found among these 3 species, suggesting a good synteny in this chromosome. These homologs all clustered on the same end of the chromosome, further reflecting that tandem repeat events took place in the expansion of this gene family. Additionally, all the homologs on chromosome 6 in potato and tomato have the highest similarity with the same gene in pepper, indicating all the homologs in these species share the same ancestor, and duplication events happened several times independently in each species.

### 2. Phylogenetic analysis of *Rpi-bib2* homologs from tomato, pepper and potato

**Fig. 2:**
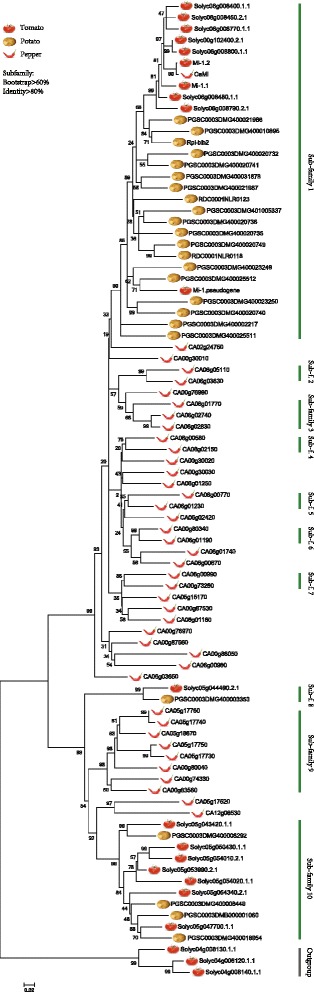
Phylogenetic tree derived from *Rpi-blb2/Mi-1.2/Cami* homologs. The maximum-likelihood phylogenetic tree is constructed by PhyML 3.1 based on the CDS sequences of each homolog

To further explore the evolutionary history of this gene family, a phylogenetic tree was constructed based on the cloned functional genes and their homologs within the 3 reference genomes. As shown in Fig. 2, genes from the same species mostly clustered together on the tree. This kind of topology further indicates that the expansion of this gene family took place separately in these 3 species after they split from their common ancestor. This tree also shows a closer homology between the tomato and potato than with pepper, coinciding with their genetic relationships on the species level.

Subfamilies were divided with an identity > 80 % and bootstrap > 60 %, with one major subfamily comprised of 30 genes, two families with 11 and 8 genes and 6 minor subfamilies, each comprised of 2–3 genes, identified (Table 2; Fig. 2). All of the 3 known *R*-genes were found in subfamily 1, the major subfamily, which includes 10 homologs from tomato, 19 genes from potato and 1 gene from pepper. Then nucleotide divergence and *Ka/Ks* were calculated within each subfamily. Nucleotide divergence varies from 0.075 to 0.150 and *Ka/Ks* varies from 0.461 to 0.795. To further compare evolutionary patterns among homologs in the largest subfamily, the divergence of genes within tomato, potato and between them was calculated. The divergence of paralogs within tomato and potato was 0.065 and 0.113, while the divergence of homologs between tomato and potato was a little higher than within species (0.122). This shows that in subfamily 1, homologs from tomato are more conserved while homologs from potato are more divergent.

**Table 2:** Nucleotide diversity, *Ka/Ks* and the number of gene conversions in each gene subfamily

Subfamily	Number of members	π	*Ka/Ks*	No. of gene conversions
Gene.subfamily 1	30	0.150	0.624	6
Gene.subfamily 2	2	0.108	0.461	0
Gene.subfamily 3	3	0.089	0.627	2
Gene.subfamily 4	2	0.128	0.584	1
Gene.subfamily 5	2	0.141	0.519	0
Gene.subfamily 6	2	0.075	0.577	0
Gene.subfamily 7	2	0.149	0.636	0
Gene.subfamily 8	2	0.075	0.582	0
Gene.subfamily 9	8	0.146	0.664	3
Gene.subfamily 10	11	0.149	0.795	2
Total/Average	64 (Total)	0.121 (Average)	0.607 (Average)	14 (Total)

### 3. Nucleotide variation in LRR regions among potato accessions

Typically, in *NBS-LRR* genes, the LRR domain is responsible for recognition of the *Avr* gene and is more divergent than the NBS domain [7]. To study the evolutionary history of the LRR domain in this gene family, 121 homologs from 9 wild and 7 cultivated potato accessions were cloned (Table 3) averaging 7.6 homologs in each accession, ranging from 5 to 10, reflecting frequent copy number variations in these accessions. Interestingly, more copy number was detected in cultivated potatoes, with 2 accessions having 10 copies and 2 having 9 copies. It might be because the cultivated potatoes are all tetraploid species, whereas most of the wild species are diploid.

**Table 3:** Statistics of the LRR regions cloned in each potato accession

Wild accession	Number of LRRs	π	*Ka/Ks*	No. of frameshift mutations
*S. demissum* 343-1	8	0.142	0.867	8
*S. demissum* 585-7	9	0.120	1.043	6
*S. microdonatum* 1169	7	0.144	0.886	2
*S. bulbocastanum* 947-2	6	0.107	0.797	0
*S. bulbocastanum* 947-1	6	0.101	1.145	0
*S. bulbocastanum* 948-5	8	0.142	0.844	4
*S. stoloniferum* 298-1	5	0.140	0.867	1
*PP10*	7	0.094	1.367	1
*S. bulbocastanum* 948-2	7	0.118	0.858	1
Average	7	0.123	0.96	2.6
Cultivated accession	Number of LRRs	π	*Ka/Ks*	
*S. tuberosum* cv. K6	9	0.155	0.833	3
*S. tuberosum* cv. 872 (T9615-1)	10	0.148	0.887	2
*S. tuberosum* cv. 873 (T9616-5)	7	0.157	0.882	4
*S. tuberosum* cv. Sarpo Mira	8	0.137	0.833	3
*S. tuberosum* cv. G18	10	0.119	0.911	5
*S. tuberosum* cv. dongnong308	9	0.141	1.037	3
*S. tuberosum* cv. kexin18	5	0.134	0.768	2
Average	8.3	0.142	0.88	3.1

To estimate the relative evolutionary rate of the LRR regions, the nucleotide divergence and *Ka/Ks* were calculated within each accession. The average nucleotide divergence of the 121 homologs in all of the 16 accessions was 0.131. The average nucleotide divergence for the wild-type and cultivated accessions were 0.123 and 0.142, suggesting that the LRR region in this resistance gene locus is polymorphic. Moreover, the divergence in cultivated accession was significantly higher than that in wild accessions (*t*-test, *P* < 0.05), which implies that the LRR domains in cultivated accession might have been diversified during the process of cultivation.

Additionally, a relatively high *Ka/Ks* ratio was found both in the wild and cultivated accessions, with 4 accessions > 1, 14 accessions > 0.8 and the other two accessions ≈ 0.8. On average, the *Ka/Ks* value of the LRR domain was higher than the whole genes from tomato, pepper and potato, indicating that the LRR domains in particular have been positively selected. When plotting nucleotide divergence against *Ka/Ks* ratio, a significant negative relationship was found (*R* = −0.61; *P* = 0.01), suggesting that the nucleotide divergence reduces as the pressure of positive selection increases. Lastly, frameshift mutations (1 ~ 2 bp indel) were annotated in each cloned LRR regions and ~30 % of the LRR regions were characterized with frequent frameshift mutations. The most frequent mutations were detected in the wild species *S.demissum*, which is a hexaploid, while the other wild accessions or genotypes had fewer frameshift mutations (0–4; Table 3). In contrast, the mutations detected in the cultivated species distributed relatively evenly. Collectively, these pseuodo-LRR domains might provide a resource or raw material for producing more new LRRs.

### 4. *LRR* regions are driven by diversified selection

**Fig. 3:**
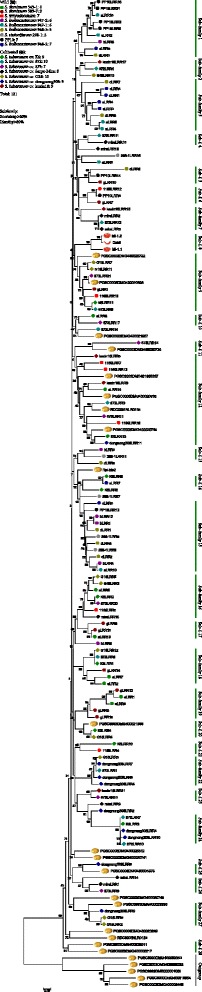
Phylogenetic tree derived from the 121 LRR regions cloned in this study and 28 LRR domains from previous studies. The 121 LRR domains were cloned from 16 different potato accessions/genotypes, 23 of the 28 LRR domains were drawn from the potato genomes and the rest 5 from previous cloned genes in potato, pepper, and tomato

To investigate the genetic relations and their evolutionary history between LRR homologs from different accessions, a phylogenetic tree was constructed based on cloned LRR regions, together with LRR sequences from potato genome and the functional genes (Fig. 3). The topological structure of the tree showed the cloned LRR regions to be scattered, suggesting that the LRR regions had a high degree of polymorphisms and varied between accessions and within accessions. Moreover, the LRR regions were not distinctly divided into two clear groups, wild and cultivated clades (Fig. 3), indicating a frequent introgression during the cultivation of potato.

However, the LRR regions from wild and cultivated accessions were not totally confused or mixed together in the tree, with most small subclades being wild-specific or cultivated-specific. Additionally, in the wild-specific or cultivated-specific subclades, the LRR regions cloned from an accession or genotype were not always clustered, illustrating the complexity of orthologous relationship among the LRR regions. It also suggests that frequent sequence exchanges may have taken place among accessions of cultivated potatoes or wild-type species.

Subfamilies were also divided based on an identity > 80 % and bootstrap > 60 %. In total, 28 subfamilies were identified, each with 2–14 members. The resistance LRRs of tomato and pepper (*Mi-1.2* and *Cami*) were clustered in subfamily 8, whereas the LRR of *Rpi-blb2* in potato was isolated. Except the resistance-specific subfamily 8 and subfamily 28 which contained only genome sequences, 8 of 28 subfamilies were wild-specific and 13 subfamilies are cultivated-specific. To further investigate evolutionary patterns of subfamilies, the nucleotide diversity, *Ka/Ks* and gene conversions were calculated and identified in each subfamily (Table 4). To obtain reliable results, subfamilies with few members (<3) were excluded from further analysis. 19 subfamilies were kept, including 10 subfamilies with ≥ 5 members. The nucleotide divergence of subfamily 8, which consists of two functional LRRs, was 0.042 with a *Ka/Ks* > 1, an indicator of positive selection. Additionally, the average number of members in 8 wild-specific subfamilies was 5.5, much higher than the number in cultivated-specific subfamilies (3.7). When comparing wild- and cultivated-specific subfamilies, the wild-specific had higher nucleotide diversity (0.060 v.s 0.020), a higher *Ka/Ks* (0.916 v.s 0.780) and an elevated rate of gene conversions events (5.88 v.s 1.83), suggesting that more abundant genetic resources could be re-used in wild species. Moreover, four complex subfamilies with 7–14 members had exceptionally high levels of polymorphisms (0.067), high *Ka/Ks* ratios (1.097) and 88 detected gene conversion events averaging 14.7 each subfamily. For the subfamilies with ≥ 5 members, the nucleotide divergence of LRR domains in paralogs, orthologs and between wild and cultivated accessions were also explored (Table 5). The average nucleotide divergence for the 10 large subfamilies was 0.053, with an average paralog divergence of 0.046, and ortholog divergence of 0.050. The highest divergence was noted between wild and cultivated accessions (0.077), which is consistent with their division distribution on the phylogenetic tree.

**Table 4:** Nucleotide diversity, *Ka/Ks* and the number of gene conversions in each LRR subfamily

Subfamily	Types of family	Number of members	π	*Ka/Ks*	No. of gene conversions
LRR.subfamily 1	Wild	9	0.060	1.400	27
LRR.subfamily 2	Cultivated	3	0.065	0.924	4
LRR.subfamily 3	Wild	8	0.075	0.989	10
LRR.subfamily 4	Cultivated	2	0.061	0.704	1
LRR.subfamily 5	Wild	3	0.086	0.989	4
LRR.subfamily 6	Wild	3	0.034	0.547	0
LRR.subfamily 7	Cultivated	4	0.051	0.532	0
LRR.subfamily 8	Cultivated	3	0.042	1.005	0
LRR.subfamily 9	Mix	9	0.048	1.147	12
LRR.subfamily 10	Mix	2	0.083	1.225	1
LRR.subfamily 11	Cultivated	2	0.153	0.818	0
LRR.subfamily 12	Mix	14	0.118	0.960	30
LRR.subfamily 13	Wild	2	0.083	1.094	0
LRR.subfamily 14	Mix	2	0.005	0.547	0
LRR.subfamily 15	Wild	11	0.052	0.807	1
LRR.subfamily 16	Mix	7	0.046	1.281	8
LRR.subfamily 17	Wild	3	0.031	0.676	0
LRR.subfamily 18	Mix	6	0.057	0.801	15
LRR.subfamily 19	Wild	5	0.059	0.822	5
LRR.subfamily 20	Cultivated	3	0.023	0.640	0
LRR.subfamily 21	Mix	2	0.128	0.718	0
LRR.subfamily 22	Cultivated	5	0.015	1.144	4
LRR.subfamily 23	Cultivated	2	0.024	0.560	2
LRR.subfamily 24	Cultivated	5	0.003	0.648	0
LRR.subfamily 25	Cultivated	2	0.079	1.440	0
LRR.subfamily 26	Cultivated	3	0.010	1.156	3
LRR.subfamily 27	Cultivated	4	0.067	0.767	0
LRR.subfamily 28	Cultivated	2	0.143	0.662	1
Total/Average		126 (Total)	0.061 (Average)	0.893 (Average)	128 (Total)

**Table 5:** Nucleotide diversity in paralogs, orthologs and between wild and cultivated accessions

Subfamily	Paralog (π)	Ortholog (Dxy)	Wild vs. Cultivated
LRR.subfamily 1	0.025	0.077	*
LRR.subfamily 3	0.058	0.061	*
LRR.subfamily 9	0.041	0.040	0.048
LRR.subfamily 12	0.130	0.113	0.129
LRR.subfamily 15	0.052	0.048	*
LRR.subfamily 16	0.017	0.047	0.050
LRR.subfamily 18	0.048	0.044	0.082
LRR.subfamily 19	0.054	0.062	*
LRR.subfamily 22	0.027	0.002	*
LRR.subfamily 24	0.004	0.001	*
Average	0.046	0.050	0.077

*represents wild-specific or cultivated-specific family

## Discussion

### 1. Copy number variation between species and between accessions

In the past years, many *R* genes have been identified and cloned in a variety of species, with the evolutionary histories of some *R* genes studied in detail [30]. Several patterns emerged in these studies. Generally, compared with other functional genes, *R* genes are always found with a higher rate of evolution. Several *R* gene families have been identified with a heterozygous evolutionary rate, with some homologs evolving faster and some homologs evolving more slowly [31, 32]. Another distinct feature is that *R* genes tend to have dramatic variations in copy number and cluster together on the chromosome [29]. In our study, the copy number of this *R* gene family was found to vary not only between species but also within species. Although the hot pepper is very closely related to the tomato and potato, the copy number of *Rpi-blb2* homologs in pepper was about 2 fold higher than tomato and potato homologs. This suggests that after the speciation of the three species, these genes may have undergone rapid copy number variations, contributing to the significant gene expansion in pepper or frequent gene loss in tomato and potato genomes.

Between wild and cultivated potato, as shown by the number of LRR regions cloned, the copy numbers also showed differences. Variations were even noted within the same species, such as among the 7 accessions of *S. tuberosum* and 4 accessions of *S. bulbocastanum* that displayed LRRs numbers from 5 to 10. Copy number variation is a common form of genome diversity that can create new genes, influence phenotypic characters and gene expression. The variation in copy number of *Rpi-bib2* homologs in different species or accessions may be a strategy to minimize fitness costs in combating pathogens or pests [33]. There are many other examples of variation in *R* gene copy number. Copy number variation (1 to 15 copies) at the *Rp1* rust resistance locus was observed in different maize cultivars [34–36]. The copy number of *RGC2* genes varied from 12 to 32 per genome in the seven lettuce genotypes [31]. Gene copy number varies between haplotypes or accessions consistent with a birth-and-death model of resistance gene evolution [37]. Some *NBS-LRR* genes are lost and new lineages evolve whilst others are retained. The main mechanism for copy number variation is unequal crossover. In our study, the variation identified among accessions within such short divergent times indicates that frequent unequal crossing over had taken place in the evolution of this gene family, and that the chromosome regions where *R* genes located may be the hot spots for recombination.

### 2. Diversifying selection in LRR regions

Till now, most of cloned plant *R* genes are identified with a nucleotide binding site and a region of leucine rich repeats, also known as *NBS-LRR* genes. The *NBS-LRR* genes are similar to *NOD-LRR* genes in mammals, which are responsible for inflammatory and immune responses [38]. The NBS domain, including ATPase and G proteins, plays an important role in the plant immune response through signal transduction, while the LRR domain, a major determinant of recognition specificity, is responsible for *Avr* gene recognition [39, 40]. LRR domains are supposed to evolve much faster and undergo stronger diversify selection than NBS domains.

In our study, we found that in the *Rpi-blb2* gene family, the NBS domains have lower divergence and are under purifying selection, while LRR domains have higher divergence and tend to suffer potential positive selection (Table 1). And a great divergence and much diversifying selection were detected in the 121 LRR regions we cloned. The high genetic abundance of LRR regions was not only found among related species, but also within species and even within accessions. These kinds of evolutionary patterns were in accordance with their functions. The NBS domain is responsible for signal transduction and thus more conserved, while the LRR domain is responsible for *Avr* gene recognition and thus part of a co-evolutionary relationship with the *Avr* gene. Pathogens evolve diverse *Avr* genes to infect plants and plants evolve diverse LRR domains in order to recognize a broad spectrum of *Avr* genes. Previous studies have indicated that tomato *Mi-1* genes from this gene family have evolved rapidly by gene duplications and frequent sequence exchanges among homologs. Here, numerous gene conversion events haven been detected in the LRR regions, promoting the generation of chimeric genes and novel resistance specificities. Additionally, ~30 % of the examined LRR domains were found to contain frameshift mutations, which is consistent with the evolutionary pattern of NBS-LRRs in *Arabidopsis* populations [41]. These LRR domains provide a potential library without fitness cost for the production of new LRRs through sequence exchange and diversifying selection [33]. Most interestingly, in the gene family we studied, LRR domains from one gene family could recognize *Avr* genes from four very different species, including nematode, aphid, whitefly and fungi, and the secret of recognizing multiple pathogens might lie in the variety of LRR domains.

### 3. Prediction of novel *R* genes

Identifying new plant *R* gene is of fundamental importance in agriculture, especially for gene families like the *Rpi-blb2/Mi-1.2/Cami* gene family, which confer resistance in more than one pathogen species. Some other *R* gene families have also been reported to include *R* genes of different species carry resistance to different pathogens, such as the *Rp1/Pi37* and *Rp3/Pc-B* gene families [42]. The maize rust resistance gene *Rp1* and rice blast resistance gene *Pi37* are orthologous and have high sequence similarity. Another two *R* genes from the *Rp3/Pc-B* gene family, the maize gene *Rp3*, which confers resistance against maize common rust, and the sorghum gene *Pc-B*, which provides resistance to root and crown rot. However, these *R* genes from grass species confer resistance to the same type of pathogen, fungi. The *Rpi-blb2/Mi-1.2/Cami* gene family in our study is an extremely special one, which includes *R* genes carrying resistance to four highly divergent organisms, including root-knot nematodes, aphids, whiteflies and the oomycete pathogen, making the presence of more resistance genes highly probable. Furthermore, members of the *Rpi-blb2* gene family, including three functional resistance genes, reside in the short arm of chromosome 6 (Fig. 1). Actually, this region is a resistance hot spot having many cloned and mapped resistance genes, including the genes coding for NBS-LRR proteins and other types of proteins, such as receptor-like proteins (RLPs). In addition to *Rpi-blb2, Mi-1.2,* and *Cami,* the tomato *RLP* genes *Cf-2* and *Cf-5*, which confer resistance to the leaf mold pathogen *Cladosporium fulvum* [43, 44], and the *Ol-4* and *Ol-6* genes, which provide resistance against the tomato powdery mildew *Oidium neolycopersici* [45], are all physically located within the same region of chromosome 6. Additionally, bacterial wilt-resistance gene *Bw-5* [46, 47], a quantitative trait locus (QTL) *Ty-1*, which confers resistance to yellow leaf curl virus in tomato [48] and QTLs with late blight and blackleg resistance in potato are also mapped to this region [15]. Obviously, this complex *R* gene region is a mine for exploiting more useful resistance resources in Solanaceae.

And the recent study found that fast evolving *R* genes in many grass species confer resistance to blast disease in rice [42]. This gives us two signs. i) Maybe it is a common mechanism that a single *R* gene family can confer resistance in multi-species. ii) The *R* genes in rapidly evolving families are most likely to confer resistance to fast-evolving pathogens. The rapidly evolving homologs of functional *R* genes are potential new *R* genes. Thus studying the evolutionary patterns of cloned functional *R* genes might helpful to predict novel *R* genes. Likewise, fast evolving members of this gene family, such as the LRR subfamily 1 or 12, exhibit high divergence and frequent sequence exchanges and thus are likely to harbor *R* genes. Clearly, the *Rpi-blb2* gene family studied in Solanaceae is very important both in scientific research and agronomy. Further investigation and comparison of the gene family in Solanaceae could provide a rich source of information for studying the evolution of *R* genes and subsequently enable the identification of potential candidate *R* genes for agricultural studies.

## Conclusions

By studying the first gene family with resistant to multiple pathogens in plant, we revealed the specialty of this gene family lies in its unusually high level of genetic abundance. Our study provided insights for the evolutionary dynamic of multiple resistance genes and also provided further evidences that maybe it is common *R* genes like this are of multi-resistance.

## Additional files

Additional file 1:
**Primers for cloning of LRRs.** (DOCX 13 kb) Additional file 2:
**Source of the 16 potato accessions used in this study.** (DOCX 13 kb) Additional file 3:
**Relative genomic positions of genes among potato (upper), pepper (middle) and tomato (lower) along chromosome 6.** (DOCX 282 kb)
