# Supplementary material for: High genetic abundance of Rpi-blb2/Mi-1.2/Cami gene family in Solanaceae
Source: BMC Evol Biol. 2015 Sep 30;15:215. doi: 10.1186/s12862-015-0493-z (PMC4590265; doi:10.1186/s12862-015-0493-z)
Supplement: Additional file 1: — Primers for cloning of LRRs. (DOCX 13 kb) [file 12862_2015_493_MOESM1_ESM.docx]

**Additional file 1. Primers for cloning of LRRs.**

| primer name | primer sequence (5’ to 3’) | primer length | product length |
| --- | --- | --- | --- |
| LRR-U | GGTAATTTGTTTCAATGAGATAGG | 24 bp | 1323 bp |
| LRR-L | CCTCCCCTCATATCTTCAGC | 20 bp |  |
