# Supplementary material for: High genetic abundance of Rpi-blb2/Mi-1.2/Cami gene family in Solanaceae
Source: BMC Evol Biol. 2015 Sep 30;15:215. doi: 10.1186/s12862-015-0493-z (PMC4590265; doi:10.1186/s12862-015-0493-z)
Supplement: Additional file 2: — Source of the 16 potato accessions used in this study. (DOCX 13 kb) [file 12862_2015_493_MOESM2_ESM.docx]

**Additional file 2. Source of the 16 potato accessions used in this study.**

| **Accessions** | **Source of material** |
| --- | --- |
| *S. demissum* 343-1 | Vivianne Vleeshouwers at Wageningen University and Research Centre |
| *S. demissum* 585-7 |  |
| *S. bulbocastanum* 947-1 |  |
| *S. bulbocastanum* 947-2 |  |
| *S. bulbocastanum* 948-5 |  |
| *S. bulbocastanum* 948-2 |  |
| *S. stoloniferum* 298-1 |  |
| *S. microdonatum* 1169 | Lijuan Zhang at Heilongjiang Academy of Agricultural Sciences |
| *PP10* |  |
| *S. tuberosum cv.* K6 |  |
| *S. tuberosum cv.* 872 (T9615-1) |  |
| *S. tuberosum cv.* 873 (T9616-5) |  |
| *S. tuberosum cv.* G18 |  |
| *S. tuberosum cv.* dongnong308 |  |
| *S. tuberosum cv.* kexin18 |  |
| *S. tuberosum cv.* Sarpo Mira | Erik Andreasson at Swedish University of Agricultural Sciences |
