# Supplementary material for: High genetic abundance of Rpi-blb2/Mi-1.2/Cami gene family in Solanaceae
Source: BMC Evol Biol. 2015 Sep 30;15:215. doi: 10.1186/s12862-015-0493-z (PMC4590265; doi:10.1186/s12862-015-0493-z)

**Additional file 3. Relative genomic positions of genes among potato (upper), pepper (middle) and tomato (lower) along chromosome 6.** Genes on each chromosome are represented by small red dots, with reciprocal best hits from the BLAST results linked by blue lines. The Rpi-blb2/Mi-1.2/Cami homologs are marked by big green dots.


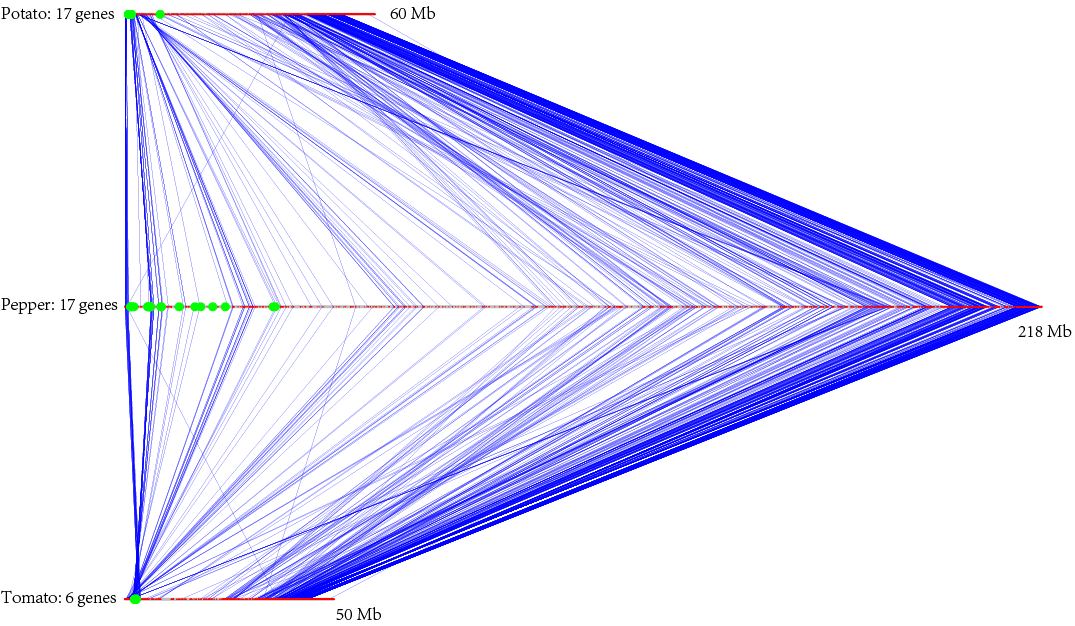

Supplement: Additional file 3: — Relative genomic positions of genes among potato (upper), pepper (middle) and tomato (lower) along chromosome 6. (DOCX 282 kb) [file 12862_2015_493_MOESM3_ESM.docx]
